# Using deep neural networks as a guide for modeling human planning

**DOI:** 10.1038/s41598-023-46850-1

**Published:** 2023-11-20

**Authors:** Ionatan Kuperwajs, Heiko H. Schütt, Wei Ji Ma

**Affiliations:** 1 Center for Neural Science, New York University, New York, NY USA; 2 Department of Psychology, New York University, New York, NY USA

**Keywords:** Human behaviour, Computational science

## Abstract

When developing models in cognitive science, researchers typically start with their own intuitions about human behavior in a given task and then build in mechanisms that explain additional aspects of the data. This refinement step is often hindered by how difficult it is to distinguish the unpredictable randomness of people’s decisions from meaningful deviations between those decisions and the model. One solution for this problem is to compare the model against deep neural networks trained on behavioral data, which can detect almost any pattern given sufficient data. Here, we apply this method to the domain of planning with a heuristic search model for human play in 4-in-a-row, a combinatorial game where participants think multiple steps into the future. Using a data set consisting of 10,874,547 games, we train deep neural networks to predict human moves and find that they accurately do so while capturing meaningful patterns in the data. Thus, deviations between the model and the best network allow us to identify opportunities for model improvement despite starting with a model that has undergone substantial testing in previous work. Based on this analysis, we add three extensions to the model that range from a simple opening bias to specific adjustments regarding endgame planning. Overall, our work demonstrates the advantages of model comparison with a high-performance deep neural network as well as the feasibility of scaling cognitive models to massive data sets for systematically investigating the processes underlying human sequential decision-making.

## Introduction

The standard approach to computational modeling in cognitive science involves handcrafting a model and specifying free parameters that are adjusted to produce behaviors consistent with empirical data^1,2^. Model predictions are then evaluated using the parameter values that achieve the best match to the data. Based on these evaluations, the model is iteratively amended to reduce remaining errors. Whether a specific change is accepted or not is usually based on model comparison techniques, balancing the tradeoff between complexity and goodness of fit. This methodology yields interpretable models because all innovations are implemented by the researcher, but it provides no guidance for when to stop searching for candidate models or what changes to try. In this pipeline, there is no way to distinguish whether the unexplained variance represents natural variability in human behavior or could be explained by a crucial change to the model. Even if it can be determined that the model needs improvement, adjustments are usually based on intuition and manual engineering.

One method for addressing these limitations is to fit deep neural networks to behavioral data. Deep neural networks make minimal assumptions about underlying cognitive mechanisms and have sufficient capacity to represent virtually any computational process^3,4^. Training a network to predict human behavior in a particular task allows the network to detect patterns in the data without requiring human understanding of these patterns. An important step is then validating that the network is indeed accurately capturing human decisions. After validation, the predictions from the network can be compared against a cognitive model’s predictions. Namely, deviations between the model and the network guide the model improvement process by highlighting situations in which the model requires novel mechanisms to explain human behavior. When there is no clear way of summarizing or pooling data across many trials, this method is more effective than simply investigating the model’s errors, which are often caused by noise that no model can explain. One potential problem with this approach is that neural networks are so flexible that they run the risk of overfitting. Regularization methods are a standard solution to overfitting in scenarios with limited data, while having access to a large data set for training can ameliorate this problem.

Consequently, neural network methods for guided model improvement have established themselves as an emerging field in cognitive science. The approach that we described in the previous paragraph is particularly useful in settings where the task is complex enough to extract additional meaningful information and when large-scale data exists to train relatively simple, feedforward network architectures. This method was pioneered to discover algorithms underlying human decision-making^5,6^ and categorization^7^. A related line of work has started to develop recurrent neural networks for automated model discovery, thus far primarily in reinforcement learning environments^8–11^. Recurrent neural networks are notoriously more difficult to train and analyze, but in turn can provide results for the simpler tasks and smaller data sets that are more ubiquitous throughout the field. Together, these approaches share the common goal of improving the process for developing cognitive models across a variety of domains.

**Figure 1:**
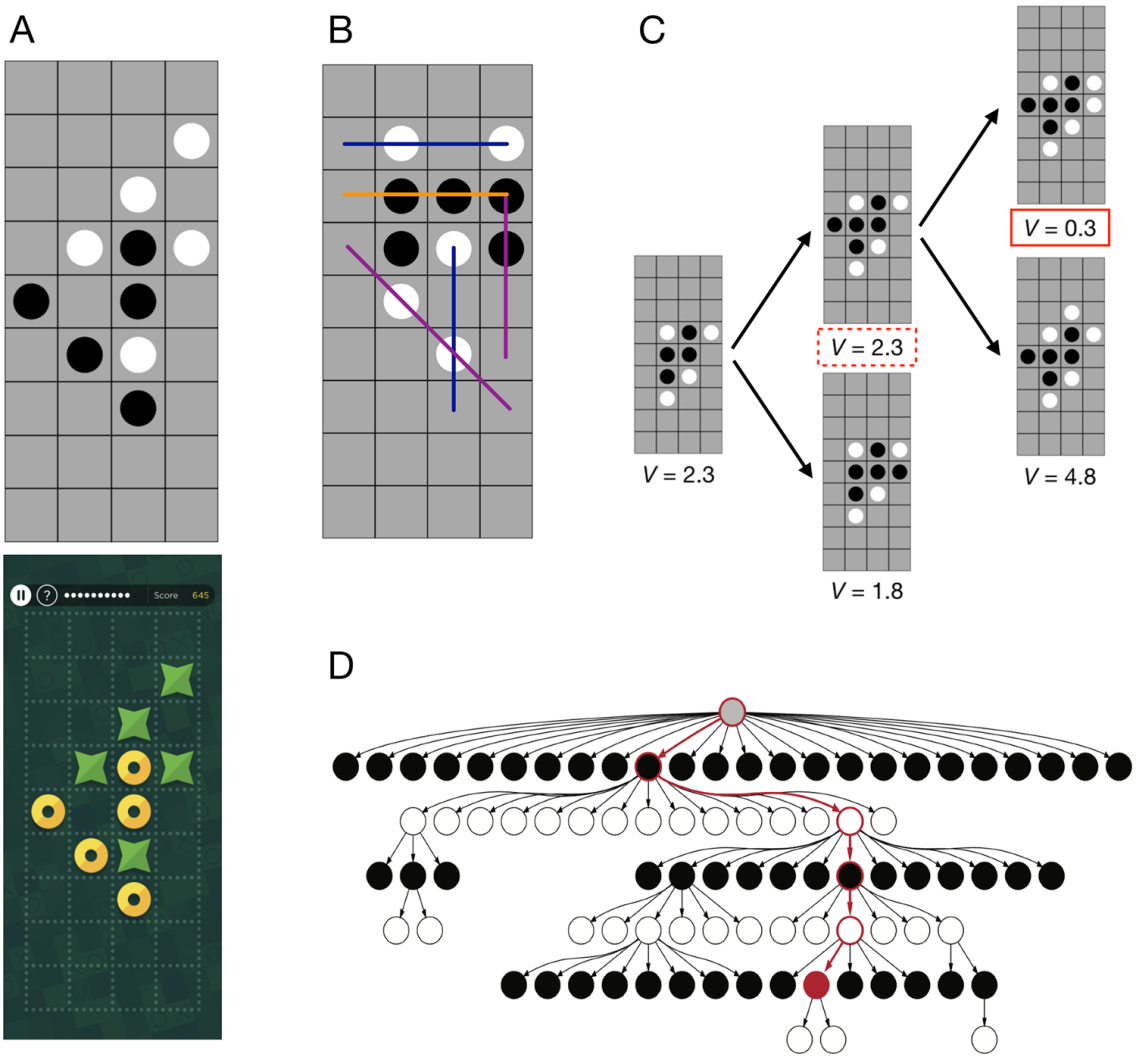
Task and cognitive model. (**A**) An example board position in 4-in-a-row in the laboratory version of the task (top) and the gamified version used on the mobile platform (bottom). Two players, black and white or yellow circles and green stars, alternate placing pieces on the board, and the first player to connect four pieces in any orientation wins the game. (**B**) Features used in the heuristic function of the cognitive model, which are intermediate patterns to winning the game. Features with identical colors are constrained to the same weights, and the heuristic evaluation is a sum over the counts of these features. The model also includes a central tendency feature and a 4-in-a-row feature. (**C**) Illustration of the heuristic search algorithm. In the root position, black is to move. After expanding the root node with two candidate moves for black and evaluating the resulting positions using the heuristic function, the algorithm selects the highest value node ($V = 2.3$) on the second iteration and expands it with two candidate moves for white. The algorithm evaluates the resulting positions, and backpropagates the lowest value ($V = 0.3$), since white is the opponent, meaning that the value in the red solid box replaces the one in the red dashed box and the root node is updated to the highest value among its children ($V=1.8$). On the next iteration, the algorithm will again expand the child node with the highest value. (**D**) Decision tree built by the model. The red nodes indicate the sequence of highest-value moves for both players. Note that different branches of the tree are evaluated to different depths.

Recently, a growing body of literature has started to examine the algorithms underlying human sequential decision-making^12–18^. Planning involves the mental simulation of future actions and their consequences in order to make a decision, but evaluating every possible course of action in real-world environments is simply intractable. Therefore, a fruitful approach has been to employ tasks with larger state spaces than are typically used in cognitive science coupled with process-level models to investigate how people plan^19^. This combination of complex tasks and models in addition to the fact that planning is an unobservable internal process limits traditional model development frameworks and makes it an ideal domain for testing more powerful methods. One such example is 4-in-a-row, a combinatorial game where players need to think multiple steps into the future to win (Fig. 1A). Additionally, human decisions have been well-described by a computational cognitive model in both laboratory and online experiments^20^. These conditions make 4-in-a-row particularly fitting for an approach to model improvement driven by neural networks: a task with many different states where the key underlying features are hard to identify, a detailed model that is already informative about human planning but can be refined further, and a very large data set for training neural networks.

Here, our main contribution is to use deep neural networks to estimate the noise ceiling, or the best fit that can be achieved on the data, relative to a cognitive model and subsequently improve that model. We begin by describing the planning task and large-scale data set as well as the heuristic search model and neural network architecture. Specifically, we emphasize the methods used to fit the model and train the neural network such that they can be compared while making use of the entire data set. Then, we show that scaling up the size of the network approaches a satisfactory upper bound on the likelihood of predicting human moves, and that the best network matches human behavior well on a variety of quantitative and behavioral measures. We investigate the residuals between the model and the network, deriving various candidate model improvements from our analysis. Namely, we implement and test three distinct mechanisms that take into account early game biases, complex interactions between model features that result in overlooked moves, and novel features in the heuristic function. Taken together, our work highlights how deep neural networks and massive data sets can be leveraged to more systematically refine cognitive models.

**Figure 2:**
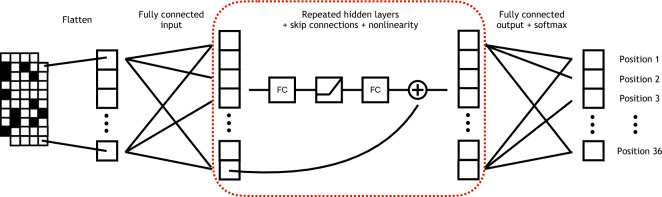
Neural network architecture. The board is represented as a $2\times 4\times 9$ tensor filled with zeros where there are no pieces and ones where there are pieces. One matrix encodes the user’s pieces, and the second encodes the AI agent’s pieces. The board representation is flattened to a 72-dimensional vector, and then passed into a series of hidden layers. Each hidden layer contains a fully connected layer, a ReLU nonlinearity, another fully connected layer, and then adds the input from skip connections (red dashed box). Finally, the fully connected output layer has 36 units and is passed through a softmax function, which yields the probability that the model assigns to the human player selecting each position of the board. In addition to varying the number of hidden layers in the network, the number of units per fully connected layer is also varied when testing different networks.

## Methods

### Task and data set

Our task is a variant of tic-tac-toe, in which two players alternate placing tokens on a 4-by-9 board (Fig. 1A). The objective is to get four tokens in a row horizontally, vertically, or diagonally. This task, which we call 4-in-a-row, is at a level of complexity that far exceeds tasks commonly used in psychology, providing rich human behavior for which computational modeling is still tractable^19^. The game has approximately $1.2\times 10^{16}$ non-terminal states, and can be leveraged to study the interplay between different reinforcement learning systems^21^, the nature of expertise^20^, or comparisons between human and machine learning^22^. Importantly, a cognitive model exists for this task, which provides a starting point for further model development.

We partnered with Peak, a mobile app company, to implement a visually enriched version of 4-in-a-row on their platform (https://www.peak.net), which users play at their leisure in their daily environment. We are currently collecting data at a rate of approximately 1.5 million games per month, and here we used a subset consisting of 82,761,594 moves from 10,874,547 games and 1,234,844 unique users collected between September 2018 and April 2019. In this version of the task, users always move first against an AI agent implementing the model of human planning that we describe in the next section, with parameters adapted from fits on previously collected human-versus-human games^20^. We partitioned the data into three sets: 90% for training (9,787,093 games), 5% for validation (543,727 games), and 5% for testing (543,727 games). The training and testing sets were used for both the neural networks and the cognitive models, and the validation set was used to monitor learning and experiment with hyperparameters for the neural networks.

### Cognitive model of human planning

**Figure 3:**
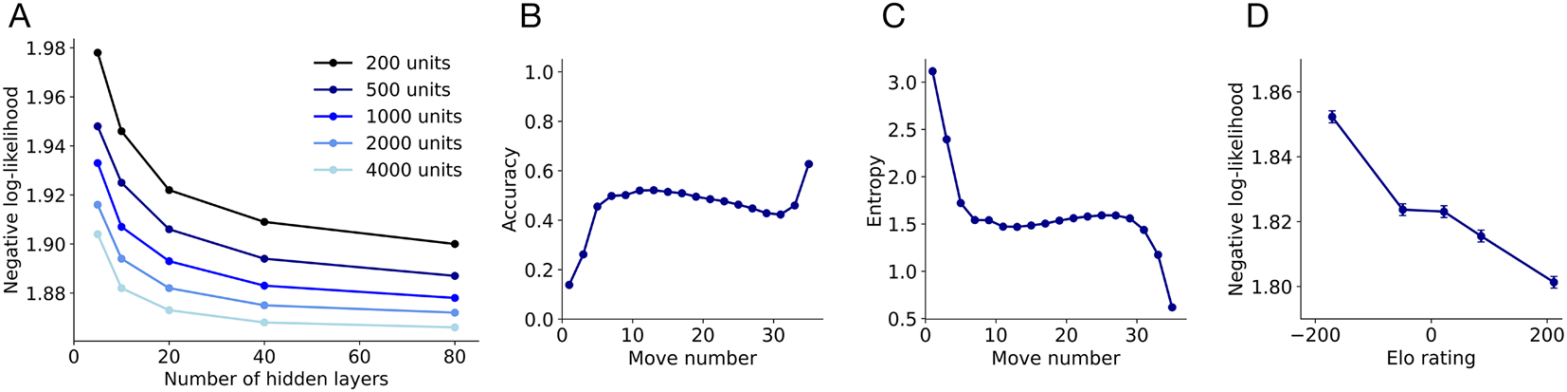
Scaling up the neural network achieves a satisfactory upper bound on goodness of fit. (**A**) Negative log-likelihood on the test data set as a function of the number of hidden layers and number of units per hidden layer in each network. (**B**) Accuracy as a function of move number for the best neural network, averaged across the test set. (**C**) Entropy of the best neural network’s output distribution as a function of move number, averaged across the test set. (**D**) Negative log-likelihood on the test data set as a function of playing strength, computed as an Elo rating (binned into quantiles).

One of the main goals of this work is to iteratively improve an interpretable cognitive model of human planning by comparing its predictions with our best neural network and subsequently testing various mechanisms inspired by this analysis. To avoid confusion, the word model always signifies this cognitive model, while the deep neural network will be referenced as the network. When we implement extensions of this cognitive model later on in the paper, we will further delineate by labeling each model. The model of interest combines a heuristic evaluation function (Fig. 1B), which is a weighted linear combination of board features^23–25^, with the construction of a decision tree via best-first search (Fig. 3C,D). Best-first search iteratively expands nodes on the principal variation, or the sequence of actions that lead to the best outcome for both players given the current decision tree^26^. To allow the model to capture variability in human play and make human-like mistakes, we added Gaussian noise to the heuristic function and included feature dropout. For each move the model makes, it randomly omits some features from the heuristic function before it performs search. Such feature omissions can be interpreted as lapses of selective attention^27^. During search, the model also prunes the decision tree by removing branches with low heuristic value^13^. In previous work, we used this model to fit behavioral data across a wide array of experiments and find robust evidence for increased planning depth with expertise^20^. As such, the model has already been subject to extensive tuning and model comparison.

While fitting the model, we estimated the log probability of a move in a given board position with inverse binomial sampling^28^, and optimized the log-likelihood function with Bayesian adaptive direct search^29^. A major technical challenge involves scaling up the fitting procedure for the cognitive model such that it makes use of the large-scale data set and is directly comparable to the neural network. To achieve this, we fit parameters for the entire training set. On each model evaluation, we evaluate the log-likelihood on 100,000 trials. We found that this yields an unbiased and sufficiently precise estimate of overall performance. We then optimized this approximate likelihood for 20 different training runs and selected the best-performing parameter set for testing. On the test data set, we ran 100 repetitions per move to estimate a log-likelihood, followed by 200 simulations in each board position to get a probability distribution over move predictions.

### Neural network training

To achieve sufficiently high performance on our data set, we constructed a deep neural network architecture that can be systematically scaled up. All of our networks take a tensor representation of the current board state and return a probability distribution for the next move over all board positions. The predictions for different board positions are independent of each other in order to match the cognitive model. We encode each board as two $4\times 9$ binary matrices. The first matrix has ones indicating the location of the user’s pieces, while the second $4\times 9$ matrix has ones marking where the AI agent’s pieces are located. Unoccupied locations contain a zero in both matrices. Thus, the input to each network is $2\times 4\times 9$, and the output of the network is a 36-dimensional vector, with each element representing a corresponding index of the board.

The architecture for our networks consists of an input layer that feeds into several hidden layers followed by an output layer (Fig. 2). The input layer flattens the $2\times 4\times 9$ board into a 72-dimensional vector and projects it to the number of dimensions used by the hidden layers with a fully connected layer. Each hidden layer consists of two fully connected layers with a rectified linear function between them and skip connections. These skip connections add the input of the hidden layer to its output without transformation, and aid in avoiding the vanishing gradient problem^30^. The output layer is a fully connected layer that projects from the dimensionality of the hidden layer to 36 units corresponding to the log probabilities for each board position. During training, we scaled the network architecture by varying the number of hidden layers as well as the number of units in each fully connected layer. In Figure S1A, we show an example loss curve for the largest network that we trained. We observed nearly identical performance on validation and test data that we did not use for training, indicating that overfitting is not an issue for our data set.

To eliminate potential predictions at squares occupied by pieces already on the board, we subtracted a large value from the output at these locations. The final softmax operator always sets the corresponding outputs to exactly 0 and normalizes the probability distribution over all open positions. This also nulls all gradients for the occupied positions such that their values are ignored for gradient backpropagation and learning during training. Prior work used convolutional networks to predict human moves in Go^31,32^, and we initially tested similar architectures in our task. However, we consistently found that the convolutional networks performed worse than the fully connected layers in preliminary training runs. Therefore, we decided to move forward using only fully connected networks.

**Figure 4:**
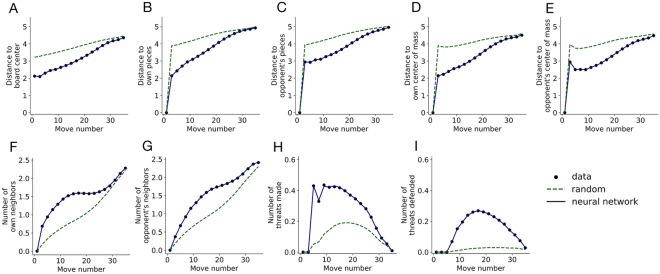
Summary statistics as validation that the neural network exhibits human-like behavior. Each statistic is averaged by move number for moves made by users (black circles), the neural network (blue lines), or a random model (green dashed lines).

## Results

### Neural network evaluation

In order to predict human behavior, we trained a total of 25 networks that varied along two dimensions: the number of hidden layers and the number of units per layer, spanning a range from 5 to 80 layers and 200 to 4000 units. We continued scaling up the networks until the log-likelihood on the test data reached a plateau, meaning that additional increases in either dimension would not lead to significant increases in performance (Fig. 3A). The largest network achieved a negative log-likelihood of 1.87 per move and a prediction accuracy of 41.71% on the test data. Additionally, this network’s log-likelihoods per move were highly correlated with the networks that are one step smaller in either direction, further supporting our conclusion that our results would not radically change with larger networks (Fig. S1B,C). Therefore, we continue to analyze the largest network in the remainder of the paper. A full specification of the networks that we trained and their performance is available in Table S1.

We then assessed whether the network convincingly captures behavioral patterns. We first considered the accuracy of the network’s predictions (Fig. 3B) and the entropy of the network’s output distributions (Fig. 3C), both broken down by move number. Intuitively, positions in the early game are harder to predict because they consist of fairly empty boards where no player can immediately win the game, and therefore result in lower accuracy and higher entropy for the network’s output. Conversely, positions in the middle and late game are much easier to predict as there are fewer alternatives and more pieces to inform decision-making, leading to higher accuracy and lower entropy for the network’s output. These positions are also more likely to contain winning moves, which lead to more stereotyped decisions. We then investigated the negative log-likelihood for the network’s predictions as a function of playing strength, computed using Elo ratings^33^, which are a standard metric for relative skill level in zero-sum games such as chess. Our network is able to more successfully capture the moves of stronger players compared to weaker ones (Fig. 3D), since unpredictable errors in gameplay are more common in the latter group. In Figs. S2–4, we show example board positions for each of the previous analyses, and in Fig. S5A,B we further analyze network accuracy as a function of number of guesses and log-likelihoods for players with varying levels of gameplay experience.

**Figure 5:**
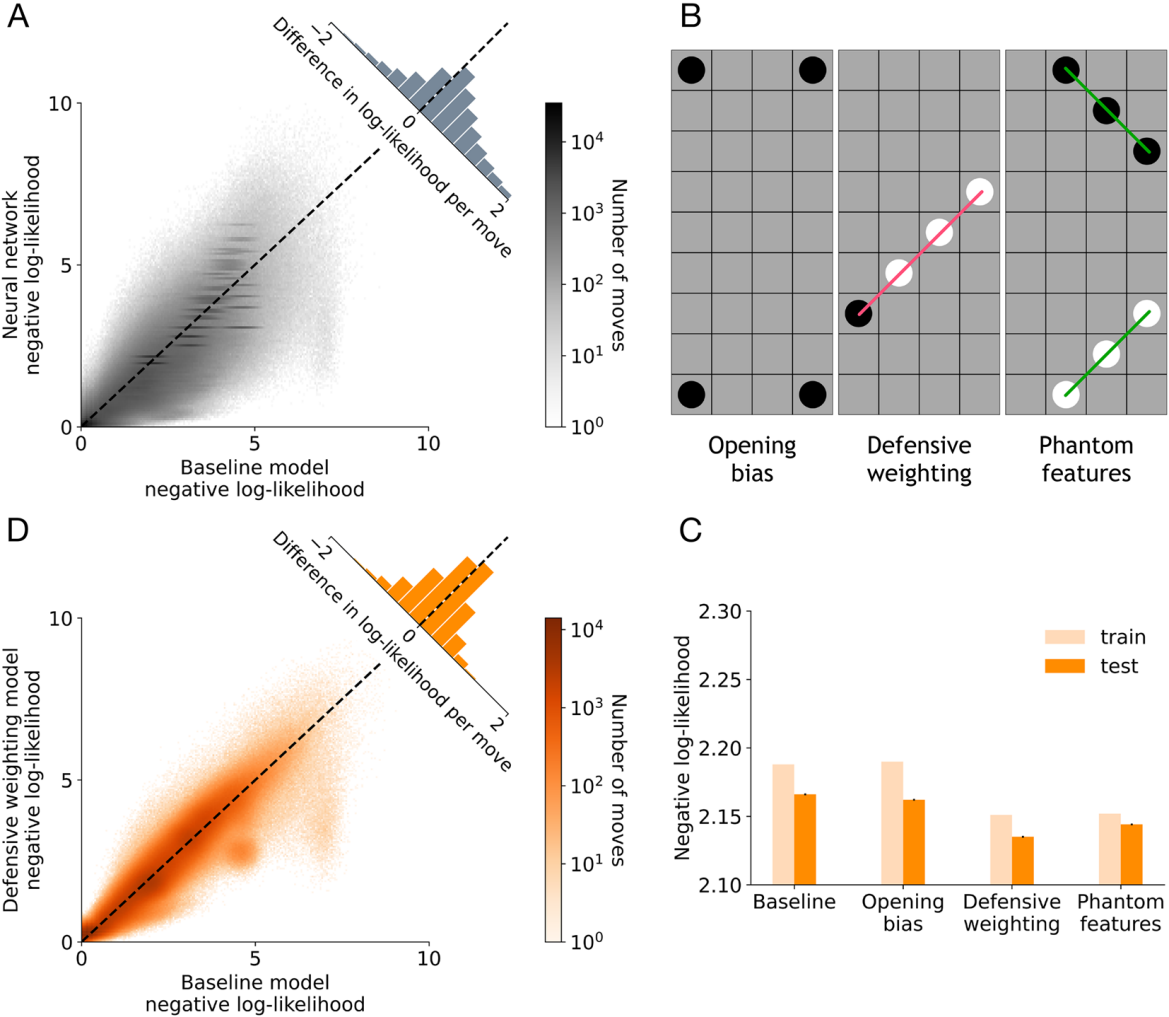
Iterating over cognitive model extensions using the neural network. (**A**) Density plot of the difference in log-likelihood per move in the test set for the neural network and baseline model. Inset is the histogram version of the same log-likelihood difference (mean of baseline minus neural network: $0.29993\pm 0.00039$). (**B**) Model extensions derived from comparing the board positions that the neural network correctly predicted and the baseline model did not. (**C**) Negative log-likelihood for each model extension for the best set of parameters across 20 different fitting runs on the training data (light orange) as well as averaged across each move in the test set for the same parameters (dark orange). Error bars indicate the standard error of the mean for the test set. (**D**) Density plot of the difference in log-likelihood per move in the test set for the defensive weighting model and baseline model (mean of baseline minus defensive weighting: $0.03097\pm 0.00022$).

Next, we computed a set of summary statistics that characterize human play in 4-in-a-row. For each move made by each user, we calculated the distance from the chosen square to the center of the board, the distance to pieces owned by that user, the distance to pieces owned by the opponent, the distance to the center of mass of that user’s pieces, the distance to the center of mass of the opponent’s pieces, the number of that user’s pieces on the 8 squares neighboring the chosen square, and the number of opposing pieces on neighboring squares. We also indicated whether with their chosen move, the user created a threat to win on the next move or parried a threat from their opponent. We computed these statistics for moves made by the network in the same positions encountered by human players and for random moves. Figure 4 shows the average of these summary statistics aggregated across all users in the test set as a function of move number. This analysis probes systematic patterns in people’s gameplay, for example a tendency to start playing near the center of the board and gradually expand outwards. For all summary statistics, people deviated considerably from random, and the neural network matched the data almost exactly. In sum, these results establish that the neural network accurately captures human decision-making in 4-in-a-row.

### Comparing the cognitive model and neural network

In terms of overall performance, the cognitive model that we have discussed so far, which we subsequently refer to as the baseline model, performed worse than the network on all measures that we tested. Specifically, the baseline model achieved a negative log-likelihood of 2.17 (0.30 more than the network) and prediction accuracy of 34.88% (6.83% less than the network) on the test data. Additionally, the network’s predicted log-likelihood per move was typically higher than that of the model ($t=322.86$, $p<2\times 10^{-308}$, Fig. 5A). The baseline model’s average accuracy per move was lower than the network’s throughout the course of gameplay (Fig. S5C), and on the summary statistics, the model deviated further from human data than the network (Fig. S6). Thus, there is room for improving the baseline model.

**Figure 6:**
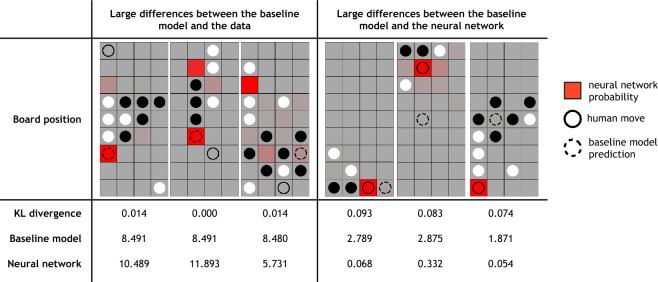
Representative residuals between the baseline model and the data (first column) and the baseline model and the neural network (second column). For each board position, we report the KL divergence between the output distributions of the model and the network, as well as the negative log-likelihood of the human move for the model and the network. The user is playing black while the computer opponent is playing white. Additionally, the red shading indicates the probability distribution of the network’s move prediction, the open circle indicates the user’s selected move, and the dashed circle indicates the baseline model’s predicted move.

Having established that there exist mechanisms that describe aspects of human behavior but were overlooked in the construction of the baseline model, our goal is to identify and implement such mechanisms. An initial attempt at this might involve the traditional model development approach, namely directly comparing the baseline model to the data. However, even with the size of our data set, most board positions beyond the early game were only encountered once by human players. Therefore, many of the 2,698,483 board positions where the baseline model predicted that a different move was more likely than the one that humans actually made represent unpredictable random human behavior rather than a failure of the model. In fact, board positions that resulted in a low log-likelihood for the baseline model were often not predicted well by the network either, and largely seemed to be human errors in gameplay such as overlooking an immediate win or making a random move (Fig. 6, first column). In short, direct comparisons between the model and data are not particularly informative.

The neural network, however, provides a viable alternate to compare the baseline model against. To do so, we used the Kullback-Leibler (KL) divergence as a measure of the difference between the output distributions of the network and model on any given board position. By pooling information across board positions, the neural network can produce a better estimate of the difference between the model and the true human policy and can thus give better guidance for model improvements. Indeed, the largest differences between the baseline model and the neural network were more interpretable than the largest differences between the baseline model and the data (Fig. 6, second column). After sorting the deviations, we manually inspected the board positions with the highest KL divergence and grouped positions together that shared identifiable features. Then, for each deviation, we implemented a change to the model to address the differences between the model and the network based on our understanding of the task and the model. We then validated that the change indeed altered the model’s predictions for the specific board positions that prompted the change. For example, when we added a new component to the model’s heuristic value function, we passed a number of board positions from the subset of deviations as input, and compared across simulations that the model now predicts the correct move where the previous iteration did not. Only after this testing procedure did we fit the amended model to the data. More specifically, we identified three mechanisms that appeared to be shared across subsets of the largest deviations between the model and the network, each leading to a new iteration of the baseline model. We implemented these cumulatively: each model contains all features of the baseline model, any prior extensions, and a new extension.

### Testing candidate model improvements

The first model extension consists of a corner bias for the opening move. The neural network comparison highlighted that users are quite likely to play in the corners in the opening, in particular in the upper left corner. There is no strategic reason for making these moves, but the network detects these preferences nonetheless. Since this pattern is especially prevalent on the first move (Fig. S5D), we added a set of parameters that can give higher value to moves being considered in each of the corners of the board (Fig. 5B, left). In terms of implementation, this mirrors the central tendency feature that is already present in the baseline model. While this was a fairly incremental model improvement in terms of negative log-likelihood on the test data as compared to the baseline model ($t=4.24, p=2.28\times 10^{-5}$, Fig. 5C), it serves as an initial proof of concept for our methodology.

The second model extension targets defending against opponent threats. In our analysis, we noticed that the model often overlooks immediate losses in favor of promising offensive moves elsewhere on the board, while both users and the network do not systematically make these errors. An example of this is shown in rightmost board in the second column of Fig. 6. This is particularly prevalent when the defensive move creates no new features for the player and the player can create multiple features for themselves closer to the center of the board. The explanation for the model’s behavior is that it assigns relatively high value to the offensive moves, causing the defensive moves to be pruned from the search tree. Thus, the defensive moves are never explored, even after the moves that the model expands preferentially are evaluated during tree search. To fix this deviation, we specify a weight in the heuristic function that explicitly recognizes immediate opponent threats (Fig. 5B, middle). With this change, the defensive moves are now no longer overlooked, as they are almost always valued highly enough to avoid pruning. As such, the defensive weighting model significantly improved in terms of negative log-likelihood on the test data from the baseline ($t=33.48, p=8.85\times 10^{-246}$) and opening bias models ($t=28.93, p=5.25\times 10^{-184}$, Fig. 5C). This further validates our proposed approach, as we were able to account for an important, more complicated mechanism that we had no prior knowledge about beforehand. Without the neural network comparison, it would have been nearly impossible to detect this detrimental interaction between pruning and the heuristic evaluation in the end game.

**Figure 7:**
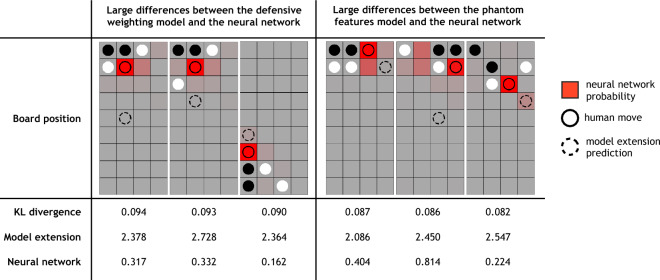
Representative residuals between the defensive weighting model and the neural network (first column) and the phantom features model and the neural network (second column). The format for the board positions is the same as for Fig. 6.

The final model extension adds phantom features. These were inspired by positions in which users preferentially play to create or defend against features that are already part of the heuristic function but do not have empty squares contained within the feature to eventually win the game. An example of this is shown in leftmost and center boards in the second column of Fig. 6. We define these in the 3-in-a-row case on the edges of the board, and include them in the model’s heuristic evaluation (Fig. 5B, right). When looking at the log-likelihoods across training runs for the phantom features model, they are fairly similar overall to the defensive weighting model, with the best parameter set that we use for testing only resulting in a difference of 0.001. This final extension did not exceed the defensive weighting model’s performance on the test set ($t=-9.87, p=5.54\times 10^{-23}$), but still improved from the baseline ($t=24.13, p=1.32\times 10^{-128}$) and opening bias models ($t=19.59, p=1.92\times 10^{-85}$, Fig. 5C). Thus, we have no evidence that people use phantom features in 4-in-a-row.

Treating the defensive weighting model as our best model variant, we show that the model’s predicted log-likelihood per move is typically higher than that of the baseline model, and that this is particularly true in moves that had a high difference in terms of log-likelihood between the models (Fig. 5D). This suggests that our added mechanisms are correctly accounting for the moves that the baseline model was initially worst at predicting. Finally, we repeated our analysis of the largest differences between the two best model extensions and the neural network (Fig. 7). As expected, this revealed that the residuals for the defensive weighting model still contain the board positions that inspired our phantom features model, whereas the residuals for the phantom features model no longer contain these and instead highlight new deviations. This suggests that the lack of improvement shown by the phantom features model is due to a tradeoff between moves in which the phantom feature weights are helping and those in which they are not. In other words, despite accounting for the desired errors that the network is able to correctly predict, the phantom features model might require an alternate implementation. It is also possible that an entirely new mechanism altogether could account for these residual board positions. A full specification of the cognitive models that we tested and their performance is available in Table S2.

## Discussion

In this paper, we trained deep neural networks to predict human moves in 4-in-a-row using a large-scale data set. We ensured that these networks estimate a reasonable upper bound on how well any model can explain human behavior by incrementally scaling up the networks and validating that any further scaling would result in marginal increases in performance. We then analyzed the best network, finding that the network captures general trends in human play. This provided us with a model that was able to predict human decisions more accurately than an interpretable cognitive model of human planning without requiring manual engineering. We then explored the positions in which the neural network was more accurate than the baseline model, leading to several candidate mechanisms for model improvement. Finally, we investigated the results from three new models that added an opening bias, defensive weighting, and phantom features, analyzing both overall goodness of fit and relative predictability compared to the neural network. Taken together, these results highlight the advantages of using deep neural networks as a guide for modeling human planning.

In comparing the neural network with our baseline model, our results suggested mechanisms that had not been previously considered and improved the model’s performance in 4-in-a-row. The defensive weighting discovery in particular is a combination of the model’s value function, forward search algorithm, and pruning mechanism that greatly affected its predictions in certain crucial positions, but would’ve been very difficult to detect without the neural network. Our findings also imply that further refinements for the model variants that we present here exist. For example, the opening bias that people display surely extends beyond just the first move and is more indicative of a faster, model-free process in the early game. Similarly, the phantom features could require a more sophisticated weighting of parameters or implementation altogether outside of the heuristic function to avoid any tradeoffs with other moves. Additionally, our approach can facilitate the generation of completely new hypotheses to explain the remaining residuals. An example in this category is reconsidering the underlying mechanism for the moves that inspired the phantom features model. Another unaddressed but consistent residual appeared for situations when the players did not start playing in the center of the board. In these games, people and the network tended to continue to play away from the center of the board in close proximity to existing pieces, while the model preferred building new central features. In sum, our approach allows for continued discovery and testing of novel mechanisms in the cognitive model. However, for the purpose of this paper, the existing set of extensions serve to primarily demonstrate the viability of guided model improvement via deep learning for complex models of human planning.

While our method provides a framework for guided model improvement with neural networks, it has several limitations. The first is that the effort of implementing, testing, and analyzing such networks might not be worth the effort if the task is simple enough. Many of the tasks that are utilized in psychology studies have a small enough state space that all substantially different situations can be investigated by hand and/or enough data is available for individual situations to make deviations between the raw data and model predictions meaningful. In such tasks, the utility of our approach is greatly reduced. Another limitation is that training neural networks requires large amounts of data. If less data is available, overfitting becomes a major concern for flexible architectures and the alternative of using less flexible network architectures implies biases in the network predictions that need to be justified. Additionally, such networks are inherently more challenging to train. Standardized tools for streamlining the neural network fitting process might alleviate these problems by reducing the burden on researchers to construct and analyze the networks. Collectively, such tool development might be worthwhile for cognitive science given the recent prominence of neural network-driven model improvement methods, but we do not provide such tools here. In terms of the method itself, a potential limitation is that we currently sort the trial-by-trial deviations and identify shared patterns manually. To automate the pattern extraction process, we could apply clustering or other machine learning methods to the board positions showing deviations between the network and the model. This would result in groups of board positions that we then inspect more closely for shared features. Another alternative might be to have 4-in-a-row “experts” who have played many games and have high estimated Elo ratings interpret the deviations between the model and neural network to reduce investigator bias. This mirrors studies in the chess literature^34–36^, and could generate new ideas for potential model improvements.

What do the mechanisms that we identify from the deviations between the model and neural network tell us about planning and human cognition? One takeaway is that people have inherent biases, meaning that they consistently prefer one out of many equivalent solutions to problems when there is no rational reason to do so. Humans display such systematic biases in many tasks^37^, and the literature on these biases and how to model them may be informative to structure the biases players show in 4-in-a-row and while planning more generally. Our model extensions also suggest that people’s heuristic functions may be more sophisticated than a simple sum of features, accounting for complex tradeoffs between pieces on the board depending on the context of the board position. Further, we observed in earlier studies that individuals seem to evaluate positions differently, as feature weights vary when the cognitive model is fit to each participant. Adjusting the heuristic function to be more human-like and account for nuanced individual differences is a challenge, but the size of the data set paired with the neural network’s predictions can guide this process. While these specific features of gameplay are tied to 4-in-a-row, they point to the interaction between heuristic evaluations and forward search, and how either of these mechanisms may change depending on the individual and context they are placed in. These are fundamental aspects of human planning, and uncovering more nuanced intuitions for how the mind navigates this process may provide principles that generalize across planning tasks.

More broadly, our work provides a framework for model construction that makes use of both deep neural networks and large-scale experiments. Cognitive science as a discipline has trended towards massive data sets collected via online studies, in part to obtain rich data in participants’ real-world environments and clarify whether results derived from constrained laboratory tasks generalize. To this end, leveraging methods from machine learning to aid in model development is a particularly important undertaking for the field. Our approach is most useful in complex tasks where comparing a model directly with human decisions is noisy due to few repetitions of any particular state. In our case, both of the previous criteria were satisfied, albeit with a cognitive model that has already undergone rigorous testing against alternatives in previous work. It is reasonable to assume that the model refinement process would be greatly expedited in situations where tedious manual adjustments derived from intuition for the task can be avoided altogether. Therefore, we argue that this method will be valuable in the development of future cognitive models of planning as well as other complex human behavior.

### Supplementary Information

Supplementary Information.

## Data Availability

The data set used throughout the current study is not publicly available per the agreement between NYU and Peak. The trained neural networks and cognitive model fits and parameters are however available upon request from the corresponding author.

## Code availability

Source code for the project is available at two separate repositories: one for for the deep neural networks and subsequent analysis (https://github.com/ionatankuperwajs/4IAR-nns) and another for the implementation of the baseline model and the model improvements (https://github.com/ionatankuperwajs/4IAR-improvements).
