# Supplementary material for: Using deep neural networks as a guide for modeling human planning
Source: Sci Rep. 2023 Nov 20;13:20269. doi: 10.1038/s41598-023-46850-1 (PMC10662256; doi:10.1038/s41598-023-46850-1)
Supplement: Supplementary file 1 — Supplementary Information. [file 41598_2023_46850_MOESM1_ESM.pdf]

Using deep neural networks as a guide for modeling human planning

Ionatan Kuperwajs[1,*], Heiko H. Schütt[1], and Wei Ji Ma[1,2]

[1]New York University, Center for Neural Science, New York, NY, United States
[2]New York University, Department of Psychology, New York, NY, United States
[*]ikuperwajs@nyu.edu

SUPPLEMENTARY INFORMATION

Large-scale mobile data

In collaboration with the mobile app company Peak (`https://www.peak.net`), we designed a large-scale study of people playing 4-in-a-row. When signing up for the app, users consented to a privacy policy, which included a provision that aggregated and anonymized data might be shared with third parties such as universities. The Institutional Review Board of New York University determined that no further consent was required and approved the research protocol as exempt.

Overall, we collected $11,529,163$ games where users always play first and the game board itself is vertically oriented and gamified. Users play at-will against a computer opponent. We filtered the data to remove games where the user times out of any move, since timing out creates games where the computer opponent plays first or makes two consecutive moves. This procedure resulted in $82,761,594$ moves from $10,874,547$ games and $1,234,844$ unique users. In order to generate the computer opponents, we made slight modifications to the cognitive model described in the main text and in greater detail later on in the supplement: (1) we used a pruning rule that keeps only the K highest-value children in each node of the search tree, (2) we added a scaling constant that multiplies the weights of features belonging to the opponent and for features of different orientation, and (3) we artificially added a delay to each computer move to simulate thinking times, which monotonically increased with the number of search iterations that the computer performed on each move. This ensured that the computer played faster in easy positions compared to hard ones. We created 7 classes of computer opponents of varying strength by specifying distinct parameter ranges derived from previous work[1] that corresponded to estimated Elo ratings[2], and matched users with an opponent on each game based on their track record of game results.

Neural network training and testing

The neural networks were all implemented using PyTorch[3]. We used stochastic gradient descent for training and reduced the learning rate if the loss associated with the validation set was stagnant for 3 epochs. The initial learning rate was set to 0.001, and we decayed the learning rate by a multiplicative factor of 0.2 at each decrease. We trained each network for a total of 10 epochs using a cross entropy loss function. Cross entropy loss is an appropriate choice for our task because it combines a logarithmic Softmax and negative log-likelihood, and is often used for classification problems where the goal is to assign weight to each of a number of classes. All layers had their biases initialized to 0 and weights drawn from a normal distribution with mean 0 and standard deviation 0.01, and we use a batch size of 128. In Figure S1A, we show example training and validation curves for the largest network. Curves for the remaining networks look similar, with a sharp improvement in the first few epochs that flattens out in later epochs, along with a minor effect caused by decreasing the learning rate.

In Figure S1B-C, we validated the network's training procedure by showing that the likelihoods are correlated between the largest network and the networks that are one step smaller in terms of either number of units per layer or number of hidden layers. In Table S1, we enumerate all combinations of networks that we trained, including the average negative log-likelihood per move and overall accuracy on the test set.

Cognitive model specification

Here we give an overview of the cognitive model of human planning, and full algorithmic details necessary for implementation are available in the paper that introduces and describes the model[1]. The model combines tree search with a feature-based value function, stochastic feature dropping, and value-based pruning.

The core component of the model is an evaluation function $V(s)$ which assigns values to board states s[4–6]. The higher this value, the more likely the player is to win from that state. We assume that people use value function approximation[7] such that

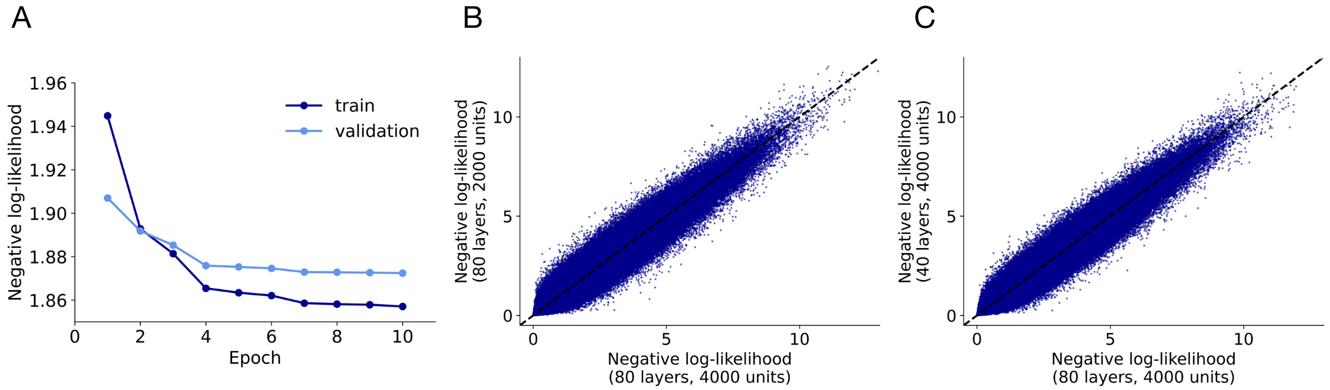

Figure S1. Neural network training procedure. **(A)** Training and validation curves over the 10 training epochs for the best network, which has 80 hidden layers and 4,000 units per layer. **(B)** Scatterplot of the negative log-likelihood for every move on the test data set between the best network and the network with 80 hidden layers but only 2,000 units per layer ($\rho = 0.99$, $p < 2 \cdot 10^{-308}$). **(C)** Same as (B), but comparing the best network with the network that has 4,000 units per layer but only 40 hidden layers ($\rho = 0.99$, $p < 2 \cdot 10^{-308}$).

Number of hidden layers	Number of units per layer	NLL	Accuracy
5	200	1.98	38.75%
10	200	1.95	39.68%
20	200	1.92	40.20%
40	200	1.91	40.63%
80	200	1.90	40.88%
5	500	1.95	39.55%
10	500	1.93	40.18%
20	500	1.91	40.73%
40	500	1.89	41.04%
80	500	1.89	41.22%
5	1000	1.93	39.93%
10	1000	1.91	40.68%
20	1000	1.89	41.03%
40	1000	1.88	41.30%
80	1000	1.88	41.41%
5	2000	1.92	40.41%
10	2000	1.89	40.98%
20	2000	1.88	41.33%
40	2000	1.88	41.49%
80	2000	1.87	41.58%
5	4000	1.90	40.69%
10	4000	1.88	41.32%
20	4000	1.87	41.55%
40	4000	1.87	41.67%
80	4000	1.87	41.71%

Table S1. All trained neural networks designated by a combination of the number of hidden layers and number of units per layer. Each network has a corresponding average negative log-likelihood per move as well as overall prediction accuracy on the test data.

their value function is a weighted linear sum of features. For this sum, we used the following 5 features: center, connected 2-in-a-row, unconnected 2-in-a-row, 3-in-a-row and 4-in-a-row. The center feature assigns a value to each square corresponding to inverse Euclidean distance from the board center, and sums up the values of all squares occupied by the player's pieces. The

Model	NLL	Accuracy
Baseline	2.17	34.88%
Opening bias	2.16	34.86%
Defensive weighting	2.13	35.23%
Phantom features	2.14	34.51%

Table S2. All tested cognitive models designated by added mechanism. Each model represents the best parameter combination on the training data over 20 runs, and has a corresponding average negative log-likelihood per move as well as overall prediction accuracy on the test data.

other 4 features count how often the associated pattern occurs on the board horizontally, vertically, or diagonally. We associate weights w_i to these features, and define the value function as follows:

$$V(s) = \sum_{i=0}^{4} w_i f_i(s, \text{self}) - \sum_{i=0}^{4} w_i f_i(s, \text{opponent}). \quad (1)$$

The evaluation function guides the construction of a decision tree with an iterative best-first search algorithm[8]. Each iteration, the algorithm chooses a board position to explore, evaluates the positions resulting from each legal move, and prunes all moves with value below that of the best move minus a threshold θ. The algorithm has a stopping probability γ, resulting in a geometric distribution over the number of iterations.

To account for variability in people's choices, we added three sources of noise. Before constructing the decision tree, we randomly dropped features at specific locations and orientations to model selective attention, which are omitted during the calculation of $V(s)$. During tree search, we added Gaussian noise to $V(s)$ at each node. Finally, we included a lapse rate λ.

Model extension specification

To iterate on the baseline model, we implemented mechanisms inspired by comparison with the best neural network. Specifically, we investigated the board positions that resulted in the largest difference in terms of predictability between the neural network and the baseline model. This resulted in three model variants, which we describe in this section. Note that each model addition is kept for later extensions. For example, the defensive weighting model has all mechanisms of the baseline model, the opening bias mechanism, and the additional defensive weighting mechanism. The negative log-likelihoods for each of these model variants on the test data set as well as their overall accuracy are shown in Table S2.

The opening bias model was inspired by early game moves that are played towards the left side of the board and the corners of the board, a phenomenon that can be corroborated by looking at the histogram of first moves made by users in the data set (Figure S5D). Therefore, we added 4 feature weights to $V(s)$, which are only active on the opening move and correspond to each of the corners of the board. This allows the model to more flexibly predict human moves that stray from the center of the board. While this addition improved the model fit, the magnitude of the effect is minor, likely because it only affects 1 out of the possible 18 moves that a player makes in any given game. A more sophisticated mechanism could extend these biases to all moves by decaying their influence throughout gameplay. Even further, humans likely use a retrospective system in early game decisions where planning is less informative[9]. If this is the case, these biases might be shaped by habit or the success of certain opening sequences in previous games. Investigating the tradeoff between prospective and retrospective decision-making is out of the scope of the current paper, but is an entire field in and of itself and integrating such a mechanism into this model would most likely improve its performance.

The defensive weighting model was inspired by situations where the baseline model failed to defend against immediate threats. In specific positions where the human player should defend against an immediate loss, the baseline model predicts that the user will instead create high-value features for themselves elsewhere on the board. This happened when the created features were valued much more highly than the removed 3-in-a-row feature for the opponent, such that the defending move was pruned from the decision tree. Both the neural network and the data did not show this pattern of oversights. To fix this, we added another feature weight to $V(s)$, which explicitly targets immediate threats made by the opponent. With this change, leaving a winning move for the opponent on the board is devalued such that the move that defends against this threat is not pruned from the tree. Additionally, we noticed that the baseline model could not overlook 4-in-a-row features during its search because it used the correct win condition to build the tree. To enable overlooking the 4-in-a-row feature, we made the detection of terminal states dependent on the 4-in-a-row feature instead and fixed the value for this feature to the arbitrary, very high value of $10,000$. This is certainly not the only possible implementation to push the model to consider defending against immediate threats, but it successfully eliminated these errors and improved the model fit. Beyond that, it is certainly plausible that people pay special attention to opponent threats as a cognitive mechanism.

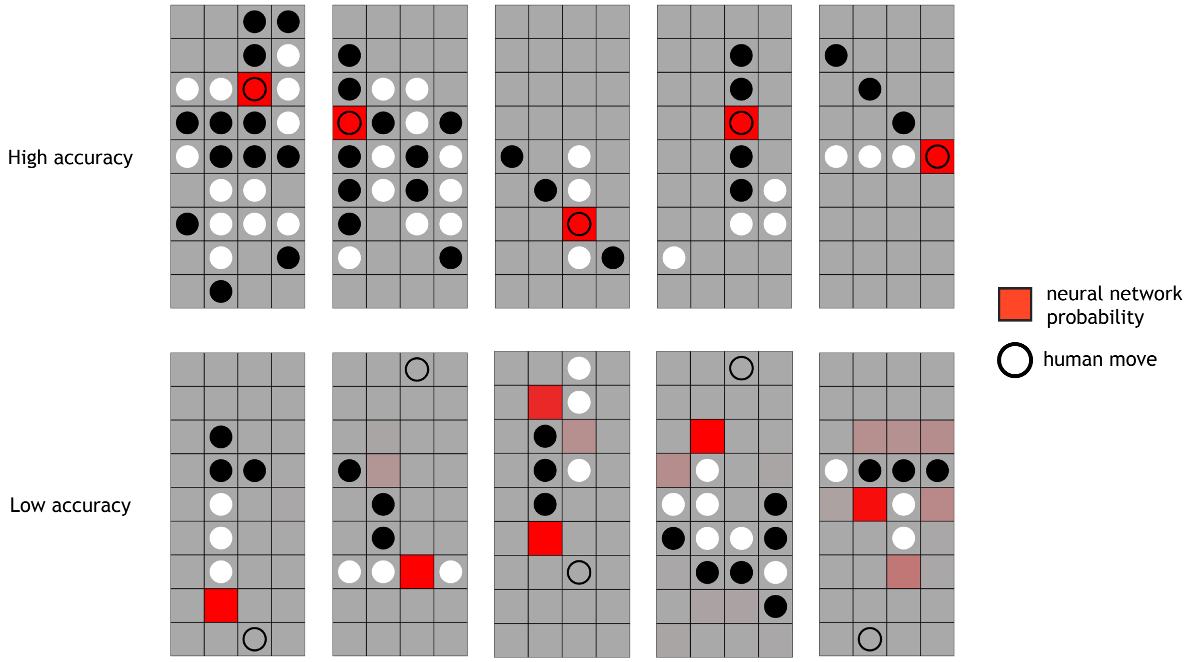

Figure S2. Example high and low accuracy board positions for the neural network's predictions. The user is playing black while the computer opponent is playing white. Additionally, the red shading indicates the probability distribution of the network's move prediction and the open circle indicates the user's selected move.

Finally, the phantom features model was inspired by board positions in which the the network and humans seemed to create or defend against 3-in-a-row features where there is no space for the final piece needed to win the game. The features in the baseline model all require that there are empty squares on the board to complete a 4-in-a-row. We enumerated the 4 3-in-a-rows that occur in the corners of the board following this pattern, and added a feature weight that scales their contribution to $V(s)$. Interestingly, this did not improve the model fit from the defensive weighting model although it did improve the predictions for the board positions that we based this extension on. This means that adding these features is causing the model to perform worse in other positions. A more general mechanism might take this tradeoff into account by, for example, checking the proximity of the piece that is being considered by the player to the rest of their own pieces. Another possibility is that these boards do not represent phantom features at all, but rather a different mechanism that can still account for these board positions. Regardless, this extension can be iterated on further to create a better cognitive model.

Model fitting

The baseline model has 9 parameters: the 5 feature weights, the pruning threshold θ, stopping probability γ, the feature drop rate δ, and the lapse rate λ. For the various model improvements, we added a few additional parameters: the 4 corner weights for the opening move, the defensive scaling weight, and the phantom features weight. For the defensive weighting and phantom features model variants, we removed one of the feature weights from the baseline model, namely the one for 4-in-a-row that is replaced by a fixed high value. Therefore, our model improvements have a total of either 13 or 14 parameters.

Unfortunately, deriving the log-likelihood analytically requires marginalization of all latent variables (i.e. which features are dropped, the value at each node and the number of iterations in the search algorithm), which is intractable. Instead, we estimated the log probability in a given board position with inverse binomial sampling (IBS)[10], which compares the data to simulated data generated from the model. IBS is unbiased but its estimates are noisy. Additionally, we cannot calculate gradients of the log-likelihood, so we optimized the log-likelihood function with Bayesian adaptive direct search[11]. In past work, the cognitive model was fit to individual users using 5-fold cross-validation to reduce overfitting. Since we wanted to make the model fits comparable with the neural network, we inferred parameters while treating the entire training set as one user, effectively eliminating any concerns regarding overfitting. To make this computationally feasible, we evaluated the log-likelihood on 100,000 trials that are randomly sampled for each evaluation. We tested both lower and higher numbers of evaluations, deciding on the value that balanced reliability of the likelihood estimates across training runs as well as fitting time. For each model variant, we ran the fitting procedure 20 different times, choosing the combination of parameters that resulted in

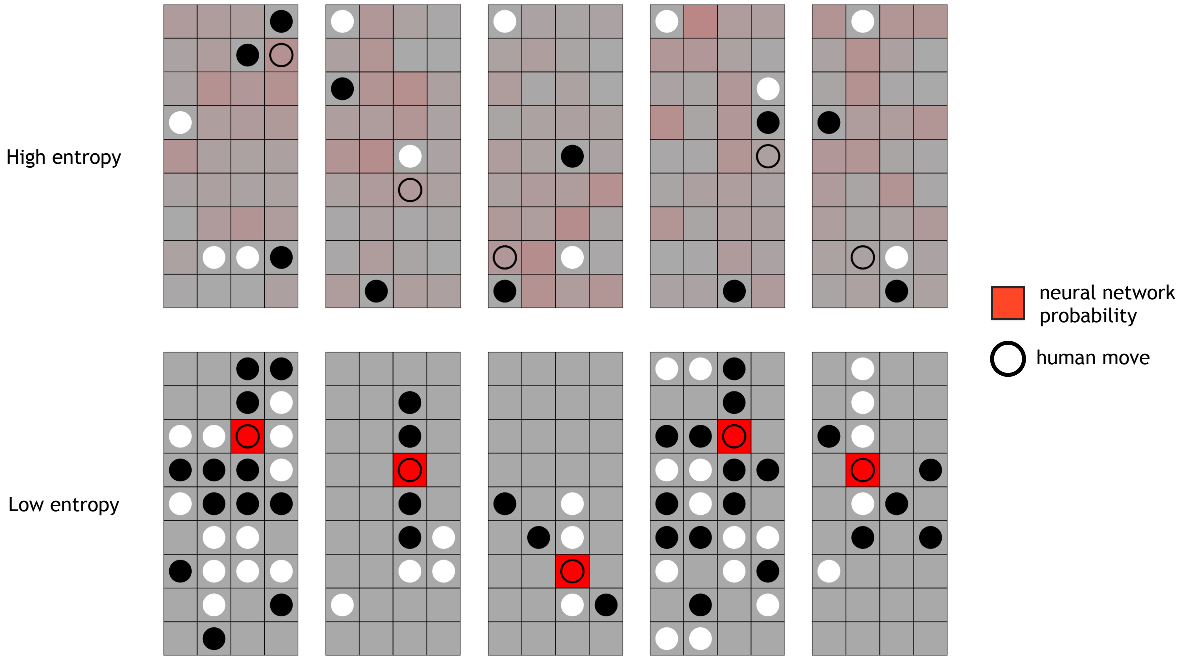

Figure S3. Example high and low entropy board positions for the neural network's output distribution. The format for the board positions is the same as for Figure S2.

the best log-likelihood. On the test data set, we then ran 100 repetitions to estimate the log-likelihoods for each move and 200 simulations in each board position to get a probability distribution over potential moves.

The fitting pipeline for both the neural network and the planning models is computationally expensive. We performed the model fits on the NYU high-performance computing cluster (Intel Xeon E5-2690v2 CPUs 3.0GHz). All of our code is implemented in parallel, including data loading and IBS. On our hardware, fitting takes anywhere from one or two days to a week depending on the size of the network or the number of evaluations used for the planning model. In this article, we trained a total of 25 neural networks and fit a total of 80 cognitive models.

Example board positions

In order to ensure that the best neural network is capturing human gameplay in 4-in-a-row with its predictions, we examined board positions sorted according to different criteria. In this section we provide a number of illustrative examples for each analysis. For the accuracy analysis, we sorted board positions by the negative log-likelihood of the network's prediction compared to the data (Figure S2). High accuracy boards were those in which there was an immediate win or loss present, or a combination of both, and the user made the same move as the network. Low accuracy boards were those in which the human made a clear error in gameplay, usually playing far away from the pieces on the board. These positions also typically include an immediate win or loss, or just a generally strong move to make that the network favors. This further serves to show that the network is approximating human behavior, minus the mistakes that we are not interested in capturing with any model. For the entropy analysis, we sorted board positions by the entropy H of the network's output distribution p_n (Figure S3):

$$H = -\sum p_n * log(p_n). \quad (2)$$

High entropy boards were those in which the network was unsure of where to play, typically consisting of only a few pieces on the board where presumably human behavior is highly variable. Even in these positions, the network assigns higher probability to squares adjacent to existing pieces on the board where people tend to play. Meanwhile, low entropy boards where the network is sure of its prediction were similar to high accuracy boards, with the network and the data agreeing on exploiting 3-in-a-rows for the player or defending against opponent 3-in-a-rows. Once again, this shows that the network is behaving in a way that aligns with our intuitions about gameplay in 4-in-a-row, confidently predicting human moves when user behavior is more stereotyped and there are more pieces on the board.

For the playing strength analysis, we estimated a user's playing strength from games against computer opponents using Elo ratings[2] (Figure S4). More specifically, we used the publicly available program Bayeselo[12]. To measure Elo ratings of all

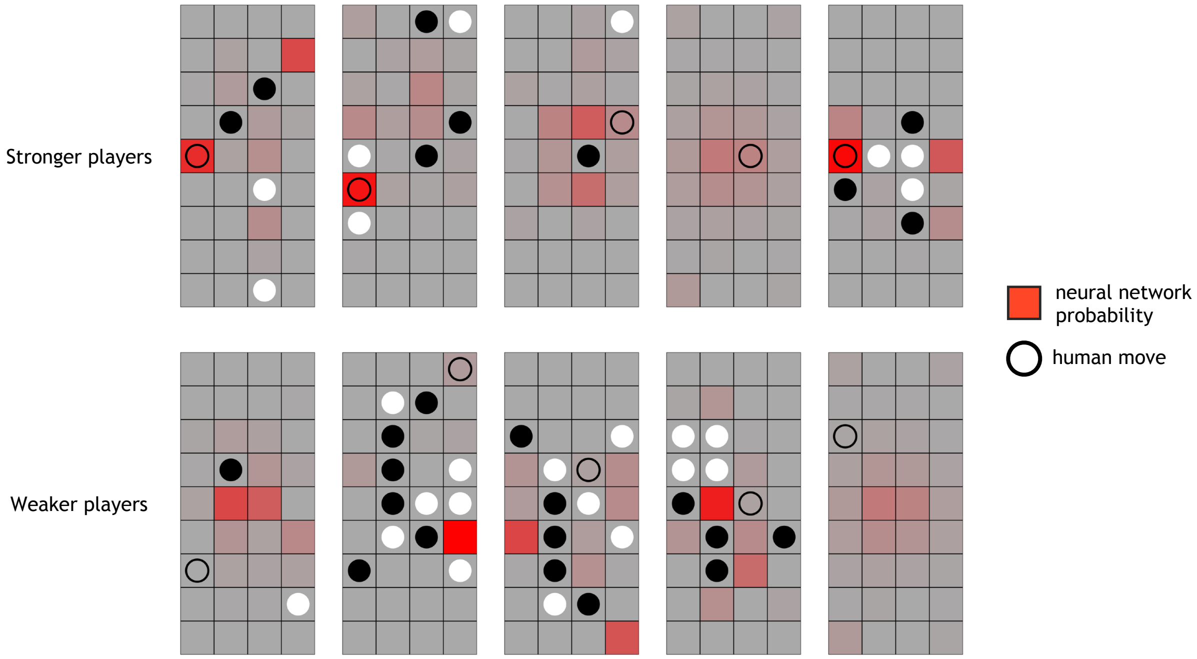

Figure S4. Example board positions played by stronger and weaker users for the neural network's predictions. The format for the board positions is the same as for Figure S2.

players against a common baseline, we ran Bayeselo on a database containing all human-vs-computer games and a simulated computer-vs-computer tournament, in which each computer plays once against every other computer, including itself. Ratings calculated for relatively few games can be statistically unreliable, so we included only players who had played at least 20 total games played in our analysis, resulting in 115,968 unique users. We used a common baseline to compute Elo ratings across all experimental data, which outputs an Elo rating for each user and AI agent in the data set that can be directly compared to one another. Moves made by players with higher Elo ratings tended to be easier for the network to correctly predict, as stronger players play more consistently and make fewer errors. For example, strong players tend to create features for themselves or block opponent features when it is rational to do so, and they play in the most common squares of the board as predicted by the network in the opening. Moves made by players with lower Elo ratings tended to be more difficult for the network to correctly predict, as weaker players make more mistakes and have more lapses in gameplay. This includes behaviors like playing far away from existing pieces, overlooking opportunities to create or defend against strong features, and playing away from the center on the first move.

Neural network validation

We conducted a few additional analyses to corroborate the neural network's performance and compare with the cognitive model. First, we validated that giving the network more opportunities to correctly predict the human move quickly converged to 100% accuracy (Figure S5A). This was indeed the case when averaged by move number, as the network starts out at its overall accuracy of 41.71% with a single guess, and converges to near perfect accuracy with only a few additional guesses. This is important as a sanity check that even if the network is wrong about the human move, the correct move is still among the top candidates. Next, we investigated the effect of experience on the negative log-likelihood of the network's predictions (Figure S5B). Number of games played is roughly correlated with Elo ratings, and repeating our playing strength analysis with experience resulted in a similar decreasing trend. Further, looking at the board positions associated with different experience levels provided additional evidence for the relationship between playing strength and experience. As expected, high experience players made moves and were predicted by the network similarly to stronger players and low experience players make errors and were predicted by the network similarly to weaker players.

In order to further compare the neural network with the cognitive model, we repeated a number of analyses from the main text with the model. One of these is average accuracy as a function of move number, where the model shows a similar trend across gameplay as the network but with a consistently lower accuracy (Figure S5C). Finally, we computed all 9 summary statistics for the model as well. Overall, the model performs similarly to the network (and in turn the data) on many of these, highlighting the fact that the model was already capturing many aspects of human play. This was expected given the large

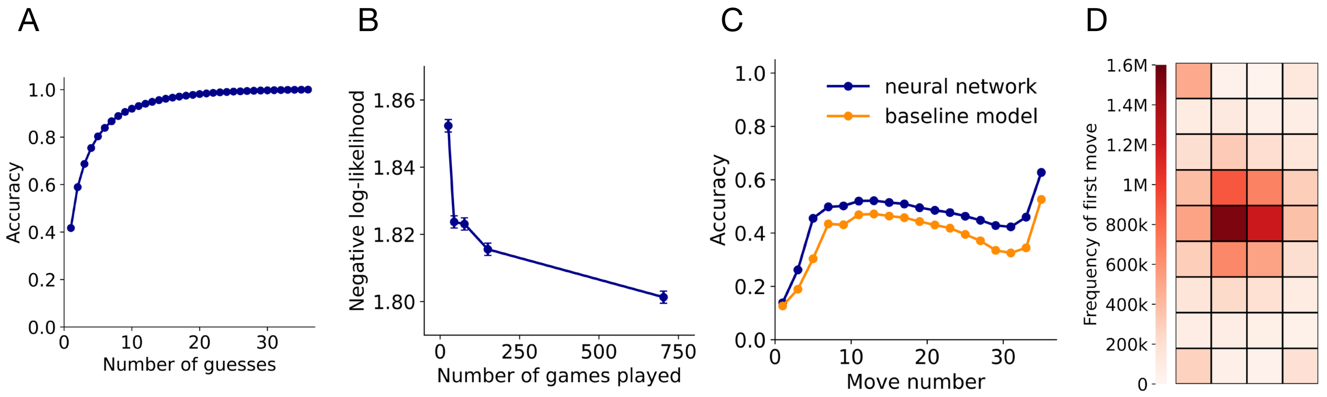

Figure S5. Additional validation that the neural network achieves a satisfactory upper bound on goodness of fit and exhibits human-like behavior. **(A)** Accuracy as a function of the number of guesses given to the neural network to correctly predict the human move, averaged across the test test. **(B)** Negative log-likelihood on the test data set as a function of the user's experience level, or total number of games played (binned into quantiles). **(C)** Accuracy as a function of move number for the neural network (blue) and baseline model (orange), averaged across the test set. **(D)** Histogram of the user's first move across all games in the data set, which the network approximates. An example board position where the network's opening move distribution is shown can be found in Figure S4.

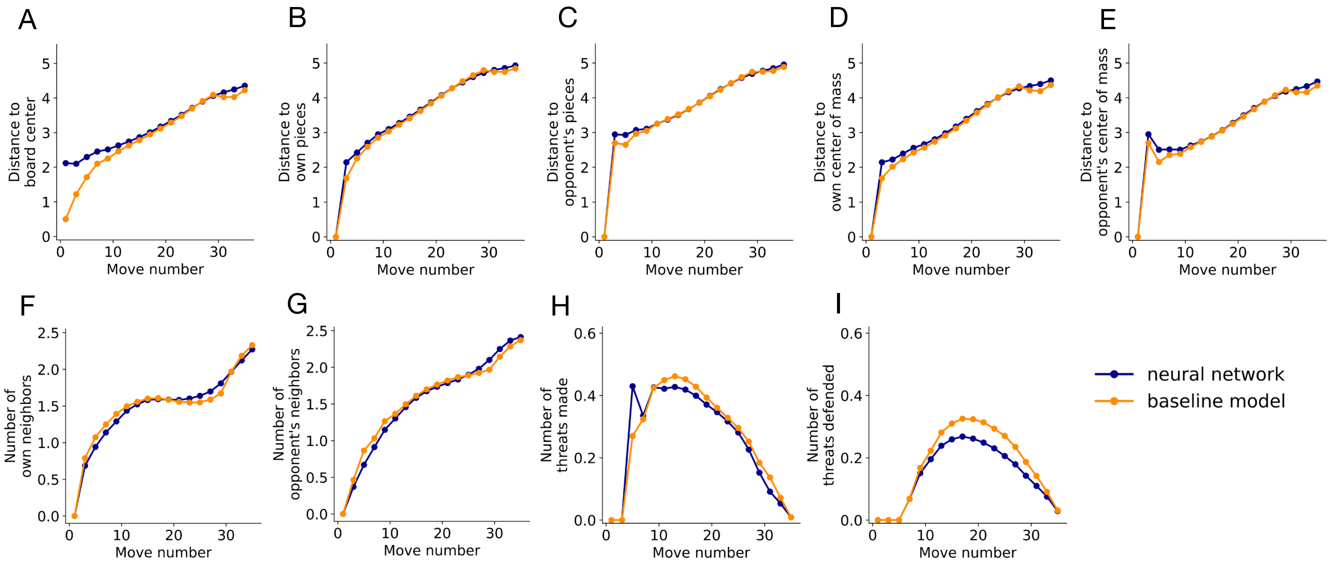

Figure S6. Comparison of summary statistics between the neural network and the baseline model. Each statistic is averaged by move number for the neural network (blue lines) and the baseline model (orange lines).

number of iterations on the model in previous work[1]. The largest deviations between the network and the model occurred in a few distinct places: (1) the distance to the center of the board in the early game where the network strayed from the center more than the model does, (2) the number of threats made where the model both overestimated and underestimated the rate at which to create 3-in-a-rows at different points in gameplay, and (3) the number of threats defended against where the model played too defensively in the middle game. These differences relate to the mechanisms we extracted from our comparison of the model and the network that we ended up implementing in our model extensions. This analysis was done in the main text by looking at board positions with high Kullback-Leibler divergence L between the network's output distribution p_n and the model's output distribution p_b on every move, defined as:

$$L = p_b \cdot \log \frac{p_b}{p_n} = p_b \cdot (\log p_b - \log p_n). \qquad (3)$$

References

1. van Opheusden, B. *et al.* Expertise increases planning depth in human gameplay. *Nature* 1–6 (2023).

2. Elo, A. E. *The rating of chessplayers, past and present* (Arco Pub., 1978).

3. Paszke, A. *et al.* Pytorch: An imperative style, high-performance deep learning library. *Adv. neural information processing systems* **32** (2019).

4. Sutton, R. S. & Barto, A. G. *Reinforcement learning: An introduction* (MIT press, 2018).

5. Bonet, B. & Geffner, H. Planning as heuristic search. *Artif. Intell.* **129**, 5–33 (2001).

6. Campbell, M., Hoane Jr, A. J. & Hsu, F.-h. Deep blue. *Artif. intelligence* **134**, 57–83 (2002).

7. Sutton, R. S., McAllester, D., Singh, S. & Mansour, Y. Policy gradient methods for reinforcement learning with function approximation. *Adv. neural information processing systems* **12** (1999).

8. Dechter, R. & Pearl, J. Generalized best-first search strategies and the optimality of a. *J. ACM (JACM)* **32**, 505–536 (1985).

9. Kuperwajs, I., van Opheusden, B. & Ma, W. J. Prospective planning and retrospective learning in a large-scale combinatorial game. In *2019 Conference on Cognitive Computational Neuroscience*, 13–16 (2019).

10. van Opheusden, B., Acerbi, L. & Ma, W. J. Unbiased and efficient log-likelihood estimation with inverse binomial sampling. *PLoS computational biology* **16**, e1008483 (2020).

11. Acerbi, L. & Ma, W. J. Practical bayesian optimization for model fitting with bayesian adaptive direct search. In *Proceedings of the 31st International Conference on Neural Information Processing Systems*, 1834–1844 (2017).

12. Hunter, D. R. Mm algorithms for generalized bradley-terry models. *The annals statistics* **32**, 384–406 (2004).
